# Sap Flow Disruption in Grapevine Is the Early Signal Predicting the Structural, Functional, and Genetic Responses to Esca Disease

**DOI:** 10.3389/fpls.2021.695846

**Published:** 2021-07-01

**Authors:** Loris Ouadi, Emilie Bruez, Sylvie Bastien, Amira Yacoub, Cindy Coppin, Lucia Guérin-Dubrana, Florence Fontaine, Jean-Christophe Domec, Patrice Rey

**Affiliations:** ^1^INRAE, ISVV, UMR1065 Santé et Agroécologie du Vignoble (SAVE), Villenave d’Ornon, France; ^2^Université de Bordeaux, ISVV, UR Œnologie, Villenave d’Ornon, France; ^3^Ecole Nationale Supérieure des Sciences Agronomiques de Bordeaux-Aquitaine, Bordeaux Sciences Agro, UMR1065 Santé et Agroécologie du Vignoble (SAVE), ISVV, Gradignan, France; ^4^Université de Reims Champagne-Ardenne, Résistance Induite et Bioprotection des Plantes (RIBP), EA 4707 – USC INRAE 1488, Reims, France; ^5^Ecole Nationale Supérieure des Sciences Agronomiques de Bordeaux-Aquitaine, Bordeaux Sciences Agro, INRAE UMR1391 Interactions Sol Plante Atmosphère (ISPA), Villenave d’Ornon, France

**Keywords:** grapevine, Esca, gene expression, sap flow, stomatal activity

## Abstract

Fungal species involved in Esca cause the formation of grapevine wood necroses. It results in the deterioration of vascular network transport capacity and the disturbance of the physiological processes, leading to gradual or sudden grapevine death. Herein, for two consecutive growing seasons, a detailed analysis of the structural (wood necrosis and leaf discoloration) and physiological parameters related to the water use of healthy and esca-symptomatic grapevines was conducted. Measurements were carried out on 17-year-old grapevines that expressed, or not, Esca-leaf symptoms in a vineyard of the Bordeaux region (France). Whole-plant transpiration was recorded continuously from pre-veraison to harvest, using noninvasive sap flow sensors. Whole-plant transpiration was systematically about 40–50% lower in Esca-diseased grapevines compared with controls, and this difference can be observed around 2 weeks before the first Esca-foliar symptoms appeared in the vineyard. Unlike grapevine sap flow disruption, structural (e.g., leaf discolorations), functional (e.g., stomatal conductance, photosynthetic activity, phenolic compounds), and genetic (e.g., expression of leaf-targeted genes) plant responses were only significantly impacted by Esca at the onset and during leaf symptoms development. We conclude that sap flow dynamic, which was related to a high level of a white-rot necrosis, provides a useful tool to predict plant disorders due to Esca-grapevine disease.

## Introduction

The vascular system is at the central core of plant functioning, and its disruption affects temporally or irreversibly plant health. This disruption may result from various factors such as occlusion of the vessels (e.g., *via* air-embolism ortyloses) or destruction of the vessels (e.g., by microbial pathogens). Among diseases that affect the plant vascular system, Esca of grapevine (*Vitis vinifera* L.) is one of the most damaging and difficult grapevine trunk diseases (GTD) to control in many vineyards worldwide, including in Europe (e.g., France, Italy, Portugal, Spain), North (e.g., California) and South (e.g., Brazil, Chile) America, Asia (e.g., Israel), and Africa (e.g., South Africa, Tunisia; Bruez et al., 2013; De la Fuente et al., 2016; Guerin-Dubrana et al., 2019).

The Esca chronic form has external symptoms generally associated with leaf necroses as atypical colors appeared due to leaf physiological changes (Christen et al., 2007). Internal symptoms imply woody tissue destruction that affects the hydraulic network of vessels and thus sap water transport (Ouadi et al., 2019). In the most severe form of the disease, i.e., the apoplectic form, complete desiccation of the grapevine can occur within a few hours or days, leading to apoplexy symptoms that cause plant death. This GTD induces high economic losses (De la Fuente et al., 2016) due to sudden grapevine death even though, in many cases, the pathological process lasts several years. During this long period, common external symptoms appear in the form of “tiger-striped” leaves on infected plants (Mugnai et al., 1999). To complicate the Esca-diagnosis of grapevines, these foliar symptoms are frequently erratic from 1 year to another and often appear long after the actual infestation of the plants. Various wood necroses are always present in dead plants, and their initial formation began years before the appearance of leaf symptoms (Mondello et al., 2018), therefore making it very difficult to precisely determine when a plant becomes infected.

Leaf hydraulic failure induced by Esca has recently been reported (Bortolami et al., 2019, 2021), and it was found that two main Esca-vascular pathogens (Phaemoniella chlamydospora and Phaeoacremonium minimum) were not directly found in leaves, although occlusions due to tyloses and gels were observed. If we look at the source of the disease, i.e., wood decay, it has been frequently reported that tracheomycotic agents, such as P. chlamydospora (Larignon and Dubos, 1997; Bertsch et al., 2013), multiple Phaeoacremonium species (Bertsch et al., 2013; Martin and Martin, 2013; Gramaje et al., 2015), and basidiomycetes species, such as Fomitiporiamediterranea, are responsible for necroses formation in woody tissues (Fischer, 2002; Fischer and González García, 2015). All these pathogens have been isolated from the woody tissues of perennial organs (Martín et al., 2019) and are able to cause wood necroses when inoculated to vines (Reis et al., 2019). At the plant level, the link between the extents of necroses, foliar symptoms, and hydraulic disruption of the whole vascular system has rarely been investigated. Recently, reduction in trunk sap flow in Esca-symptomatic grapevines was measured, using thermal dissipation sap flow sensors. This experimental approach showed that the maximum transpiration rate of grapevines expressing Esca-foliar symptoms was 30% lower than that of healthy grapevines (Ouadi et al., 2019). In this study, the reduction in sap flow was related to a high level of necroses, especially the white-rot necrosis caused by *F. mediterranea*.

Based on these observations, it is clear that quantifying the reduction in transpiration in relation to leaf-level physiological mechanisms in Esca-diseased grapevines seems to be relevant in order to understand how fungi can ultimately cause plant death. Previous reports showed that early physiological alterations, such as dysfunctions of the photosynthetic apparatus, induction of a defense mechanism, and accumulation or alteration of phenolic compounds, were observed 1 week before Esca-leaf symptoms appeared in apoplectic grapevines (Fontaine et al., 2016; Calzarano et al., 2018). However, several aspects of the link between internal (wood necroses, vascular dysfunction) and external symptoms (leaf discoloration and physiology) remain unclear, and so is the link between the disruptions of the photosynthetic apparatus and changes in the activities of primary metabolism genes. Another key point would be to understand whether or not infested grapevines respond differently to climatic factors prior to any external visible Esca-leaf symptoms that appeared so we can predict the prevalent conditions for the disease to develop.

The present study aimed to investigate sap flow disruption across the main phenological stages of the grapevines before and following visible Esca-disease symptoms. We then related those changes in grapevine transpiration to environmental conditions and also to plant physiological (loss in whole-plant conductance and stomatal conductance) and chemical (nitrogen balance index, phenolic compounds) responses and to the expression of specific primary metabolism genes involved in physiological dysfunctions caused by Esca. These grapevines were planted 17 years ago in a commercial vineyard whose plants have been monitored for Escasince plantation. First, we observed the extent and the type of wood necrosis in the trunk and cordons of healthy and Esca-symptomatic grapevines that had been physiologically monitored for 2 years. Secondly, we analyzed the operating efficiency of sap-conducting vessels with grapevine-adapted thermal sensors placed directly around small and young (1-year-old) stems from healthy and Esca-diseased grapevines. This heat balance method used to measure sap flow does not require the intrusion of a needle-shaped probe in the xylem and thus maintains the integrity of the hydraulic system intact. By associating internal and external Esca-symptoms in the same study, specific physiological changes were compared between Esca-symptomatic and healthy grapevines.

## Materials and Methods

### Study Site and Plant Material

The study site was located within the Château Luchey-Halde estate (a vineyard owned by Bordeaux Sciences Agro) located in Mérignac, Bordeaux region, France (latitude: 44.8218, longitude: −0.6291). The study was conducted on 17-year-old Cabernet Sauvignon grapevines (*Vitis vinifera* L.), grafted on 10-114 MGT rootstock, and planted in a sandy clay soil at a density of 8,000 grapevines/ha. The grapevines were pruned in winter, using a double Guyot training system. To maximize the amount of sunlight that reaches the grapes, the canopy was mechanically trimmed, and leaves regularly thinned throughout the growing season at a height of 120–130 cm and a width of 50–60 cm. The experimental plot was chosen because it had been surveyed for the presence of Esca-foliar symptoms since its plantation in 2000. Only grapevines that had previously expressed chronic Esca-foliar symptoms at least two times over a period of 3 years between 2014 and 2016 were considered symptomatic. The control plants never expressed the Esca-foliar symptoms. In order to acquire qualitative and quantitative characterizations of the physiological disruptions caused by Esca, 16 asymptomatic and 16 foliar-symptomatic grapevines, without variability in terms of vigor, were selected. Grapevines in the experimental plot were classified in the following categories: S = Esca-symptomatic grapevines with pronounced symptoms related to 20–100% disease severity; and A = asymptomatic grapevines that never expressed Esca-foliar symptoms in the previous years. Grapevines that had showed Esca-leaf symptoms in the previous years but did not maintain a continuous Esca-foliar expression throughout the monitoring period were discarded from the analysis. The grapevines that showed less than 20% disease severity were not included in the S category because the symptoms were generally located in basal leaves, which have a low impact on yield quality and quantity.

The survey was conducted over a 2-year period in 2017 and 2018, from a fruit set to berry ripening. Climatic conditions were characterized with agrometeorological data acquired from a weather station, located on a neighboring plot less than 200 m from the monitored grapevines. Air temperature, wind speed, relative humidity (RH), photosynthetic active radiation (PAR), vapor-pressure deficit (VPD), and rainfall were recorded on a half-hour basis, and data transferred daily. During the monitoring period, leaf-level physiological and chemical measurements were conducted on both Esca-symptomatic and asymptomatic leaves. Whole-plant water transport was continuously recorded in 12 grapevines, composed of six control (A) and six Esca-diseased plants (S), by installing sap flow heat sensors around the stem. The phenological stages chronology were assessed through weekly observations, using the international BBCH scale as a reference (Biologische Bundesantalt, Bundessortenamt und Chemische Industrie; Eichhorn and Lorenz, 1970). In 2017, the physiological monitoring of grapevines was carried out between July 2, which corresponded to the phenological stage 77 on the BBCH scale (bunch closure), and September 17 (harvest). The first Esca-foliar symptoms were observed on July 19 at stage 83 (mid-veraison). In 2018, the physiological monitoring was carried out between June 10 at the phenological stage 69 on the BBCH scale (end of flowering) and September 17 (harvest). The first Esca-foliar symptoms were observed on July 12 at stage 77 (bunch closure).

In order to follow the evolution of the physiological response in the Esca-diseased grapevine to the onset of foliar symptoms, Esca notations were performed on the 32 monitored grapevines, whenever leaf level measurements were carried out. The prevalence of Esca-foliar symptoms was calculated at the end of each year, when all symptomatic grapevines had expressed foliar symptoms. The prevalence of Esca-foliar symptoms corresponds to the ratio between the numbers of grapevines that expressed Esca-foliar symptoms at the time of the notation and the total number of Esca-symptomatic grapevines.

### Leaf-Level Physiological Parameters

Stomatal conductance (g_s_) of the 32 monitored grapevines was measured, using a Li-Cor 1600 steady state porometer (Li-Cor Corp., Lincoln, NE, United States). Only sun-exposed and recently fully expanded leaves were used for measurements, with a minimum of two replicates per cordon. Leaf numbers 5 and 7 counted down from the top of the shoots were systematically measured, with the first leaf having a central vein longer than 3 cm being considered as leaf number 1. The measurements were conducted two times a week between 10:00 a.m. and 12:00 a.m., corresponding to daily g_s_ maximum values. When Esca-foliar symptoms became visually apparent, measurement frequency was increased to every 2 days. However, because of the high occurrence rate of phytosanitary treatments in the vineyard, which is common during the vegetative and pre-harvest period in the Bordeaux wine region, a total of 11 and 14 days of measurements were possibly achieved in 2017 and 2018, respectively.

### Leaf Nitrogen and Phenolic Compounds

The Dualex 4 Scientific (Force-A, Orsay, France) was used to monitor grapevine leaf nitrogen status and assess leaf phenolic compounds. The Dualex apparatus is based on chlorophyll fluorescence excitation screening by UV-absorbing compounds of the epidermis as described by Goulas et al. (2004). The Dualex leaf-clip measured four optical indices: chlorophyll (Chl), epidermal flavonols (Flav), nitrogen balance index (NBI) that is the ratio of the two previous indices, and epidermal anthocyanin (Anth). Those optical indices were systematically performed, following porometer recordings between 12:00 a.m. and 01:00 p.m. on the same no. 5 and no. 7 leaves on which g_s_ was measured.

### Leaf Area Index

Leaf area index was measured nondestructively for each monitoring season, using normalized difference vegetation index (NDVI; Johnson, 2003), which, for vineyards, has been shown to accurately represent the spatial variability of a leaf area (Hall et al., 2002; Towers et al., 2019). The experimental plot was mapped by Fruition Sciences (Bordeaux, France) using high-resolution aerial imagery acquisition. Each year, two plane-flight campaigns were performed to determine individual NDVI values of grapevines equipped with sap flow sensors, using a spatial resolution of 10-cm ground sampling distance. The first flight was carried out in June at stage 75 (berries pea sized), when no Esca-foliar symptoms had yet appeared on infected grapevines. A second flight was performed in August at stages 83–89 (berry ripening) when the prevalence of Esca-symptoms reached 80%, and most foliar discolorations had been expressed. The QGIS software (QGIS Development Team 2013) was used to georeference grapevines equipped with sap flow sensors and extract their individual NDVI values through image processing. Leaf area index of each monitored grapevine was calculated, using the linear relationship between NDVI and grapevine leaf area index (LAI) described by Johnson (2003).

### Stem Sap Flow and Grapevine Water Use

Sap flow (F, g.h^−1^) was measured on 12 selected grapevines, including six Esca-diseased and six control plants. The first day of sap flow measurement started on July 9, 2017, at the beginning of veraison (stage 81 on the BBCH scale). In 2018, sap flow sensors were reinstalled under the same conditions, starting June 19 at stage 75 on the BBCH scale (berries, pea sized). During both years, sensors were maintained on grapevines until harvest (September) and thus followed the progressive development of Esca-foliar symptoms. A total of six measurement subplots were installed in the experimental plot. At each subplot, two grapevines, one healthy and one Esca-diseased, located in the same row and distant from a maximum of 10 m, were equipped with sap flow sensors.

Stem flow sensors, 10 mm in diameter (EXO-Skin™Sap Flow Sensor; Dynamax, Inc., Houston, TX, United States), were provided by Fruition Sciences and installed on one vine cordon corresponding to the wood of the year n-2 to avoid irregular basal trunks or ground temperature gradient effects. Dynamax sap flow sensors design consisted of a heating sleeve wrapped around the grapevine stem. The sleeve flexibility ensured a snug fit on every stem section and enabled sap flow sensors to provide heat uniformly and radially across the inner wood. In addition, as the entire stem section is heated, this heat balance method can be applied even if a sap flow trajectory through the stem is tortuous. Thermal grease was used to optimize contact between the sensor and the wood before applying thermal and waterproof protection around the sensors. In addition, trunks, cordons, and stems were wrapped with reflective insulation, using an aluminum sheath to minimize direct solar irradiance. Maintenance operations were performed every 2 weeks to assess the status of the whole installation and prevent any damages that could be caused by a continual application of heat under low flow rates conditions. The signals emitted from the sap flow sensors were scanned every 30 s and the data computed and stored in SAPIP data loggers (Dynamax, Inc., Houston, TX, United States) every 15 min. Arduino open-source microcontroller boards, equipped with a data logger shield, were connected to the SAPIP to send the data collected by sap flow sensors directly to Fruition Sciences servers and perform real-time assessments of the measurement quality on each site. Coolterm software was used to store the sensor output data in text file format. An autonomous power supply system, consisting of a 12 V slow-cycle battery (Eversol, 90 Ah), a 50-W solar panel, and a charge regulator (Stecasolsum, 6 A), was used to continuously supply the sensors, the SAPIP data loggers, and the arduinos.

The sap flow rate (F) was calculated, using the stem heat balance (SHB) method. The continual heat applied to the stems enables the measurement of F by monitoring the thermal regime of the stem. This method involves solving the heat balance over a stem section by applying constant or variable power to continuously heat the tissue (Cermák et al., 1973; Baker and Van Bavel, 1987; Peressotti and Ham, 1996). A constant power of 2 V was applied to each sensor, and temperature gradients (∆T) were measured by three thermocouples mounted within the heat sensor. The thermocouples continuously measured the variations in temperature differences from the heater strip to the stem, the ambient, and into the sap flow. The temperature of the heating sensor was, therefore, maximum when the value of the sap flow is zero and decreases as the sap velocity increases since an increasing amount of heat is being evacuated by convection. This temperature difference corresponding to the amount of heat transported in the moving sap was then used to compute stem sap flow (F, g.h^−1^). The SAPIP system used in the experiment recorded the signals and with a well-accepted energy balance formula (Baker and Van Bavel, 1987). Sap flow measurements were also calculated on a ground area to obtain whole-plot transpiration (using stem density to convert F to kg.m^−2^.day^−1^ equivalent to mm.day^−1^) and on a leaf area basis (using LAI to convert F to mmol.m^−2^.s^−1^). From 1 day to the next, variations in stem water content of the grapevines are generally neglected as water storage compartments are refilled at night (Meinzer et al., 2009), and the daily plot transpiration was assimilated to the total sap flow cumulated over 24 h (Domec et al., 2009).

### Necroses Image Analyses

Following the physiological monitoring period (September, 2018), grapevines equipped with sap flow sensors were uprooted and cut longitudinally. One-half of each trunk was photographed for quantification of necrotic wood, which was, in comparison to apparently healthy wood, darker brown in color and varied in texture from hard (necrotic tissue) to soft and spongy (white rot). The severity of each necrotic area was assessed from the photographs, using the image-analysis software ImageJ (NIH, United States^1^), and necroses were evaluated according to the percentage of trunk area infected as described by Liminana et al. (2009).

### Gene Expression: RNA Extraction and Real-Time RT-qPCR Analysis

Among grapevines equipped with sap flow sensors, four Esca-diseased and four control plants had their leaves sampled five times throughout the 2018 growing season to investigate the expression of genes involved in physiological dysfunctions caused by Esca. Care was taken to only sample leaves from the same height of the canopy at which both stomatal activity recordings and leaf chemical measurements were made. For each sampling time, healthy, sun-exposed, and fully expanded leaves were collected from the middle section of each cordon for both control and Esca-diseased grapevines. With regard to the BBCH scale, leaf samples were collected two times at stage 75 (berries, pea sized), corresponding to T0 and T1 when few Esca-foliar symptoms were visible; one time at stage 77 (bunch closure), corresponding to T2 when the prevalence of Esca-foliar symptoms attained 20%; two times at stage 85 (veraison), corresponding to T3 and T4, when the prevalence of Esca-foliar symptoms reached 80%. Among the four Esca-diseased grapevines that were sampled, two plants continuously expressed Esca-foliar symptoms during the season. Starting on T1, a total of four Esca-symptomatic leaves were collected during each sampling phase. Specifically, leaves collected from each sampled vine were immediately frozen in liquid nitrogen then stored at −80°C. RNA extraction was performed according to the protocol described by Reid et al. (2006). A total of 20 samples were extracted for each modality (Esca-diseased and control grapevines). For Esca-symptomatic leaves, a total of 10 samples were extracted, and their analysis was performed in comparison with the healthy leaves collected on the same symptomatic grapevines. The leaves were ground in liquid nitrogen to a fine powder, using a pestle and mortar. The leaf powder was then added to an extraction buffer (20 g.ml^−1^), preheated to 56°C (300 mM Tris HCl, pH 8.0, 25-mM EDTA, 2-mM NaCl, 2% CTAB, 2% polyvinylpolypyrrolidone (PVPP), 0.05% spermidine trihydrochloride, and 2% β-mercaptoethanol added extemporaneously). The mixture was mixed vigorously and incubated in a water bath at 56°C for 10 min under regular stirring. An equal volume of chloroform: isoamyl alcohol (24:2, v/v) was added and then centrifuged at 3,500 *g* for 15 min at 4°C. The following RNA extraction steps were conducted, using the SpectrumTM Plant Total RNA Kit protocol where RNA was captured in a unique solution onto a binding column, which effectively prevents polysaccharides as well as genomic DNA from clogging. Residual impurities and the most residual genomic DNA were removed by DNase treatment and with wash solutions. Purified RNAs were eluted in RNase-free water. The amount of RNA obtained was measured at 260 nm and 280 nm by spectrometry (NanoDrop 1000 Spectrophotometer, France), while its integrity was assessed by electrophoresis on an agarose gel and by passage over a Bioanalyzer (Agilent technology, France). Total RNAs (150 ng) were reverse-transcribed, using verso cDNA Synthesis kit (Thermo Fisher Scientific, Waltham, MA, United States). Real-time polymerase reaction (PCR) was performed, using Absolute Blue qPCR SYBR Green (Thermo Fisher Scientific, Waltham, MA, United States), in a CFX96 real-time PCR detection system (Bio-Rad, Hercules, CA, United States). The thermal profile was 10 s at 95°C (denaturation) and 45 s at 60°C (annealing/extension) for 40 cycles. The specificity of PCR amplification was checked, using a heat dissociation curve from 65 to 95°C, following the final cycle. PCR efficiency of the primer sets was calculated by performing real-time PCR on serial dilutions of cDNA. For each experiment, PCR reactions were performed in duplicate, and two independent experiments were analyzed (Mondello et al., 2019). A mean quantification cycle (Cq) value was calculated for each gene sampled, and values greater than 30 were not considered and discarded. Results were normalized with two reference genes (EF1-α and 60SRP, Table 1), and relative gene expression (RE) was determined with the two^-ΔΔCq^ methods (Livak and Schmittgen, 2001), using CFX Manager 3.0 software. For every sample, ΔΔCq was the ΔCq difference between the two samples. Expression of genes-encoding enzymes involved in the carbohydrate metabolism (αA, βA, and SucS2), the photosynthesis activities (RbcL, SBP, and psbP1), the photosynthetic electron transport (Fd), the plant defense mechanism (PR6), and the stress response system (HSP-α, PIP2.2) was tracked by quantitative reverse transcription-PCR (qRT-PCR), using the primers listed in Table 1.

**Table 1:** List of primers used for quantitative reverse transcription (RT-qPCR).

Gene	Encoding	Primer sequences 5' – 3' (forward/reverse primer)	GenBank or TC TIGR accession number NCBI accession
EF1-α	Elongation factor 1-alpha	AACCAAAATATCCGGAGTAAAAGA	XM_002284888.3
		GAACTGGGTGCTTGATAGGC	
60SRP	60S ribosomal protein L18	ATCTACCTCAAGCTCCTAGTC CAATCTTGTCCTCCTTTCCT	XM_002270599.3
αA	Alpha amylase	AGCTTGTGGACTGGGTGAAA GAGGCCCTCCATTTGAGTCC	XM_002285177.3
βA	Beta amylase	TACCATACACTATCACACCCCATAATCCAGCCTTATCAAACC	XM_002265662.3
SucS2	Sucrose synthase 2	TGTGGGCTTCCTACATTTGCT	XM_002271494
		CCTTCTGGCATCGTTCAAAGA	
RbcL	Large subunit of RuBisCO	AATTTTTCCTCCACGGCGATA ATCTGCGCCCGCCTTTATA	TC57584
SBP	Sedoheptulose-7-biphosphatase	TGCCAACCAGCTCCTATTTGA	XM_002263013.3
		TCAACTGGGCCTCCCATGT	
psbP1	Oxygen-evolving enhencerPsbP subunit of photosystem II	GCTGACGGAGATGAAGGTGG	AY222741
		AACCAAAATATCCGGAGTAAAAGA	
Fd	Ferredoxin	TGTGGATCAGTCTGACGGGA CTCCTCCTCCTTGTGGGTCT	XM_002269581.3
PR6	Serine proteinase inhibitor	AGGGAACAATCGTTACCCAAG	AY156047.1
		CCGATGGTAGGGACACTGAT	
HSP-α	Alpha crystalline heat shock protein	TCGGTGGAGGATGACTTGCT	XM_002272382.3
		CGTGTGCTGTACGAGCTGAAG	
PIP2.2	Plasma membrane instrinsic protein aquaporin	TACACAAAAGCCCAAAGCTAACA	AF141900
		CAACTAAAAACCCACAACACCC	

### Statistical Analysis

Statistical analyses were performed in R software (R Core Team, 2016). As described in Ouadi et al. (2019), differences between Esca-leaf symptomatic and asymptomatic grapevines were tested, using analyses of variance (ANOVA), which were performed for each physiological parameter. The normality of the residuals of the variables was tested, using the Schapiro–Wilk test. When the validation tests were not significant, a nonparametric test was used (Kruskal–Wallis). All the tests were taken as significant when *p* < 0.05.

## Results

### Grapevine Phenological Stages and Prevalence of Esca Symptoms

**Figure 1:**
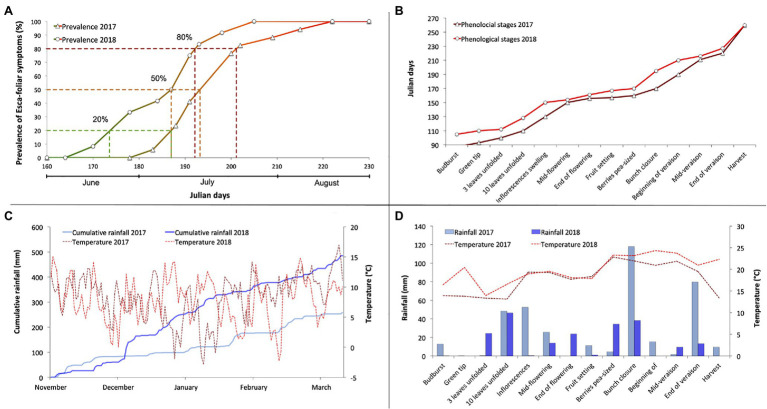
Characterization of the sanitary, phenological, and climatic context of the 2-year physiological monitoring period, showing **(A)** the evolution of the prevalence of Esca-foliar symptoms in 2017 and 2018, **(B)** the chronology of the main phenological stages in 2017 and 2018, **(C)** the comparison of winter cumulative rainfall and temperatures during the 2017 and 2018 winter seasons, and **(D)** the comparison of rainfall and temperatures during the 2017 and 2018 growing seasons.

The year 2017 was marked by a cold and dry winter until January then by a mild and rainy weather in February and March, which led to an early bud burst (Figures 1C,D). Two successive episodes of frost at the end of April affected a small portion of the experimental plot but did not damage the grapevines selected for the physiological survey and the determination of Esca symptoms (Figure 1C). Nevertheless, a phenological delay was detected due to the low temperatures at the beginning of the growing season (Figures 1B,C). The summer of 2017 was exceptionally dry but did not cause water stress, especially on account of the major rain events of June when grapevines achieved bunch closure (Figure 1D). In comparison, 2018 was rather a wetter year than 2017, as the cumulated winter rain between November 1 and March 1 was about 385 mm compared with 200 mm the previous year (Figure 1C).

During the 2-year physiological monitoring, field notations were performed on the 16 Esca-symptomatic grapevines (S1 to S16) and the 16-asymptomatic or control plants (A1–A16) to assess Esca-foliar symptoms (Table 2). During both growing seasons, grapevines LAI values of Esca-symptomatic and healthy grapevines, calculated using NDVI recordings, did not differ significantly and varied between 2.3 and 2.8 m^2^ m^−2^ (Supplementary Figure S1). Out of those 32 surveyed grapevines, changes in symptoms expression occurred on eight plants. Control plants presenting Esca symptoms and Esca-diseased grapevines that did not continuously express Esca-foliar symptoms were discarded. It should be noted that the frequency of appearance of foliar symptoms from 1 year to another increased proportionally with the risk of apoplexy. Among the 16 symptomatic grapevines that were monitored, three died by apoplexy at the end of the experiment. From the evolution of Esca appearance in 2017 and 2018 (Figure 1A), three thresholds were used to follow the progressive development of Esca-foliar symptoms: 20% (beginning of Esca-foliar expression), 50% (when half of the symptomatic grapevines expressed symptoms), and 80% (when most of the grapevines expressed symptoms). Grapevine phenological stages were consistent between 2017 and 2018 (Figure 1B). In both years, most Esca-foliar symptoms appeared during berry ripening as the prevalence of Esca-foliar symptoms reached 80% at day of the year (DOY) 193 in 2018 and at DOY 202 in 2017 (Figure 1A).

**Table 2:** Notation table presenting the status of the 16-year-old Cabernet-Sauvignon cultivar (*Vitis vinifera* L.) that underwent a 2-year physiological monitoring in 2017 and 2018.

Grapevine references		2014–2016	2017	2018
Asymptomatic grapevines	A1	✓	✓	✓
	A2	✓	✕	✓
	A3	✓	✓	✓
	A4	✓	✓	✕
	A5	✓	✓	✓
	A6	✓	✓	✓
	A7	✓	✓	✓
	A8	✓	✓	✓
	A9	✓	✓	✓
	A10	✓	✓	✓
	A11	✓	✓	✓
	A12	✓	✓	✕
	A13	✓	✓	✓
	A14	✓	✓	✓
	A15	✓	✓	✓
	A16	✓	✓	✓
Symptomatic grapevines	S1	✕	✕	✓
	S2	✕	✕	✕
	S3	✕	✕	✕
	S4	✕	✕	✕
	S5	✕	✓	✕
	S6	✕	✕	✓
	S7	✕	✕	✓
	S8	✕	✕	✕
	S9	✕	✕	✓
	S10	✕	✕	Apoplectic
	S11	✕	✕	✕
	S12	✕	✕	✕
	S13	✕	✕	✕
	S14	✕	✕	Apoplectic
	S15	✕	Apoplectic	Apoplectic
	S16	✕	✕	✕

Esca-foliar symptoms observations are represented by (✕) while healthy grapevines are represented by (✓). Grapevines with references ranging from A1 to A6 and S1 to S6 were equipped with sap flow sensors and were uprooted at the end of the experiment.

### Necrotic Wood Ratio

**Figure 2:**
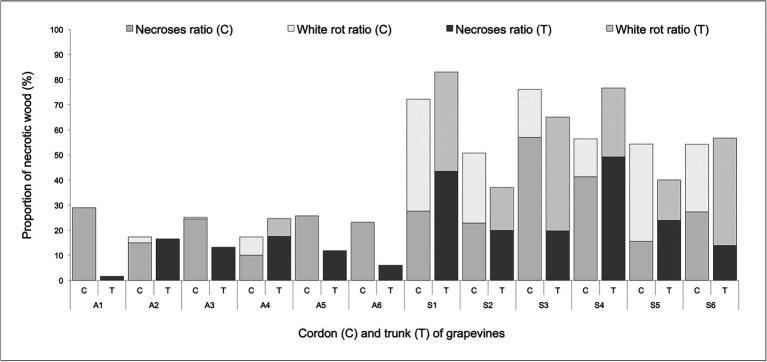
Percentage of necroses and white rots in the trunk wood (T) and cordons (C) of asymptomatic (A) and symptomatic (S) Cabernet Sauvignon grapevines.

The largest amount of white-rot necrotic wood was found inside the trunk and cordons of grapevines that expressed Esca-foliar symptoms (Figure 2). Regardless of the tissue considered in these symptomatic plants, 15–50% of the total necrotic area was made of a white-rot necrosis. Within asymptomatic grapevines, there was a very low proportion of white-rot, and less than 30% of the total wood surface was necrotic. Among the control plants, A2 and A4 had a total necrotic wood ratio close to the other asymptomatic grapevines but still expressed Esca-foliar symptoms in 2017 and 2018 (Table 2). White rots were found in their trunks and cordons, but the proportion of white rots in A2 and A4 accounted for less than 10% of the total wood surface and remained significantly lower when compared with other Esca-symptomatic grapevines (Figure 2).

### Grapevine Water Use and Plot Transpiration

**Figure 3:**
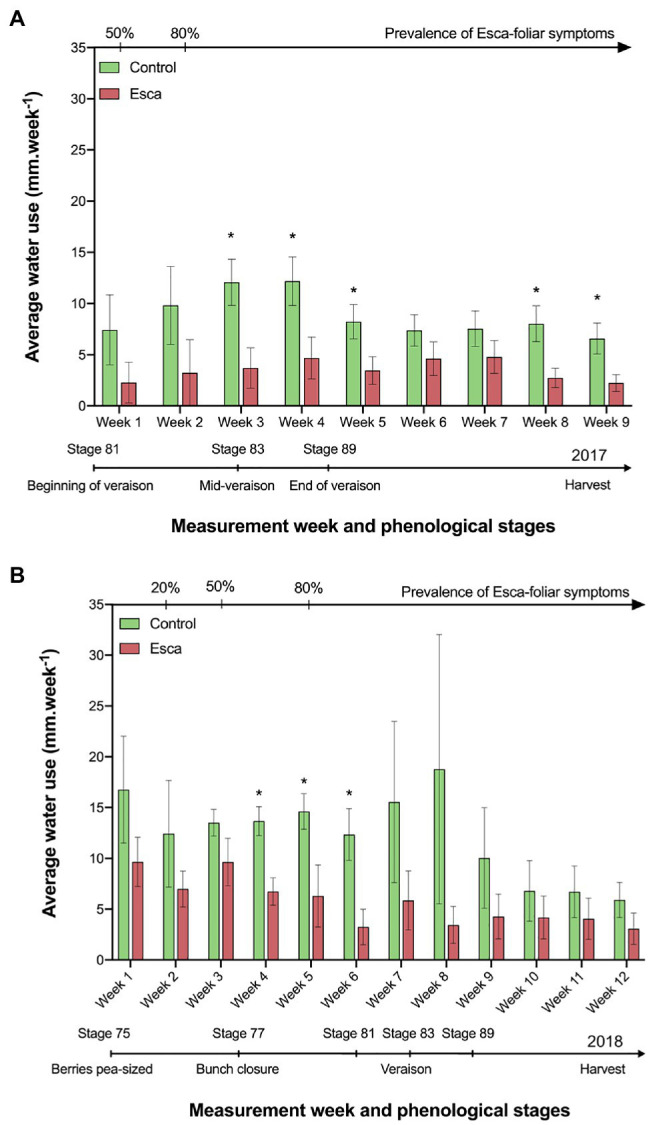
Evolution of grapevine water use in control and Esca-symptomatic Cabernet Sauvignon grapevines. Comparison of the average grapevine water use between Esca-diseased and control grapevines in 2017 **(A)** and in 2018 **(B)**. Key-growing phenological stages and the prevalence of Esca appearance are also represented below and above the horizonal axes, respectively. Error bars represent SE. Stars (*) indicate a significant difference (at *p* < 0.05) in water use for a given week between asymptomatic and symptomatic grapevines.

In 2017, sap flow data were acquired continuously from stage 81 on the BBCH-growing scale (beginning of veraison, i.e., mid-July) to harvest in September. During that period, temperatures did not exceed 27°C, and the mean maximum daily vapor-pressure deficit (VPD) remained around 2.5 kPa (Supplementary Figure S2B). For clarity, we are mostly presenting transpiration data on a ground-area basis. However, because no difference in LAI between treatments was apparent, patterns in grapevine water use either on a leaf- or ground-area basis were similar between symptomatic and asymptomatic plants. Water use of healthy plants was significantly higher compared with plants that expressed Esca-foliar symptoms (Figure 3A). Although the same trend remained over the entire monitoring period, significative differences (*p* < 0.05) were measured between mid-veraison (stage 83) and harvest. The average water use in Esca-symptomatic grapevines became two to three times lower compared with control plants. For example, at mid-veraison (week 3), the average water use in healthy plants was 12 mm.week^−1^ vs. 4 mm.week^−1^ for Esca-diseased plants, which corresponded to a daily water use of about 2.14 L.d^−1^ for healthy vines against 0.71 L.d^−1^ for grapevines that expressed Esca-foliar symptoms.

Similar trends were recorded in 2018 (Figure 3B), but significant differences were measured earlier in the season between bunch closure (stage 77) and the beginning of ripening (stage 81). In 2018, the data recording phase started at stage 75 (berries, pea sized, i.e., mid-June) and was marked by higher temperatures and VPD values than in 2017. Sap flow reached a midday plateau, which was maintained for about 6 h between 10:00 a.m. and 04:00 p.m. Sap flux increased shortly after sunrise, reached a peak by 2:00 p.m., and decreased in the late afternoon (Supplementary Figures S1A,B). For a given health status, no significant correlation (*R*^2^ < 0.31, *p* > 0.1) could be established between rainfall, temperature or VPD, and the average daily or weekly water use of grapevines (data not shown).

**Figure 4:**
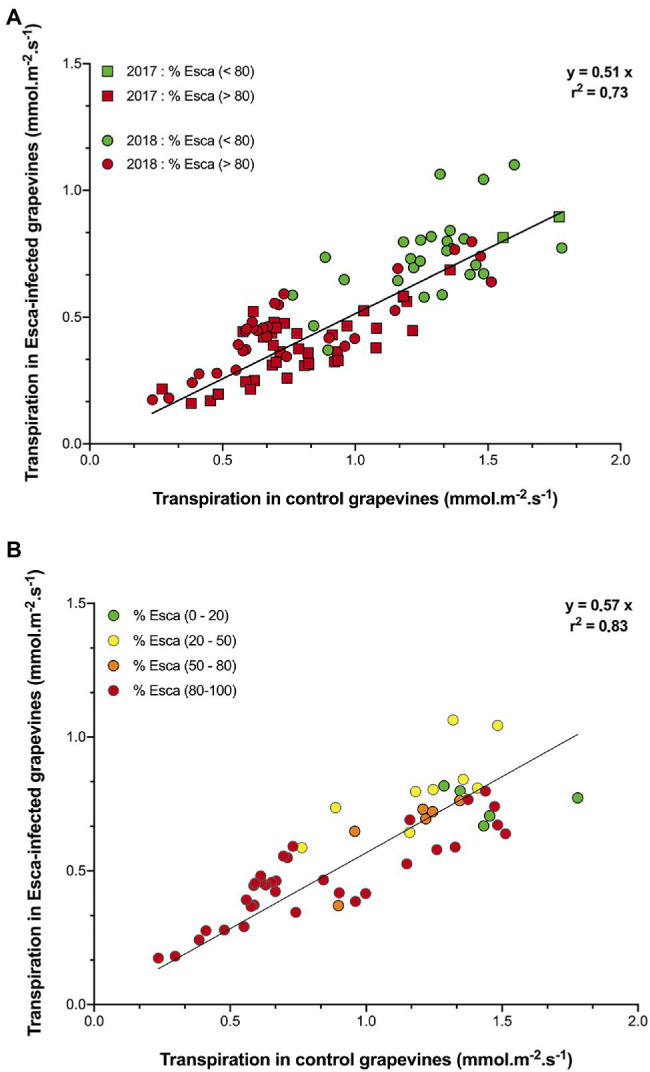
Comparison of transpiration in asymptomatic and symptomatic Cabernet Sauvignon grapevines. Data represent transpiration values under non-limited light conditions, i.e., measured between 10:00 a.m. and 4:00 p.m. In **(A)** data were separated to reflect days “before” (green symbols) and “after” (red symbols) the prevalence of Esca-foliar symptoms reached 80% in 2017 (squares) and 2018 (circles). In **(B)** data from 2018 were separated to reflect days with different prevalence levels of Esca symptoms: 0–20% (green symbols), 20–50% (yellow symbols), 50– 80% (orange symbols), and 80–100% (red symbols).

Regardless of the prevalence of Esca-foliar symptoms, the analysis of transpiration dynamics according to the health status of grapevines revealed two distinct kinetics. Although hourly transpiration recorded in Esca-diseased and control grapevines showed a similar trend, the maximum transpiration rates (measured around midday when the light was not limiting) of grapevines that started to develop Esca-foliar symptoms were significantly lower (*p* < 0.05) than in control plants (Figure 4A). On average, leaf-based transpiration ranged from 0.2 to 1.7 mmol.m^−2^.s^−1^ corresponding to ground-level transpiration of 0.3 to 2.7 mm per day. The daily transpiration measured in Esca-symptomatic grapevines was about 40 to 50% lower than in healthy plants (*p* < 0.001). Before and after the expression of the Esca-foliar symptoms, this downward trend remained the same (Figure 4B). In 2018, the effect of an extensive leaf pruning to allow more sunlight on the grapes a few weeks before the harvest was indicated by a sharp decline in transpiration (i.e., week 9 in Figure 3B, or day 230 in Supplementary Figures S1A,B). However, even after this large reduction in LAI, the difference in transpiration rates between asymptomatic and symptomatic vines remained significant.

### Leaf Stomatal Conductance, Photosynthesis, and Phenolic Activity

**Figure 5:**
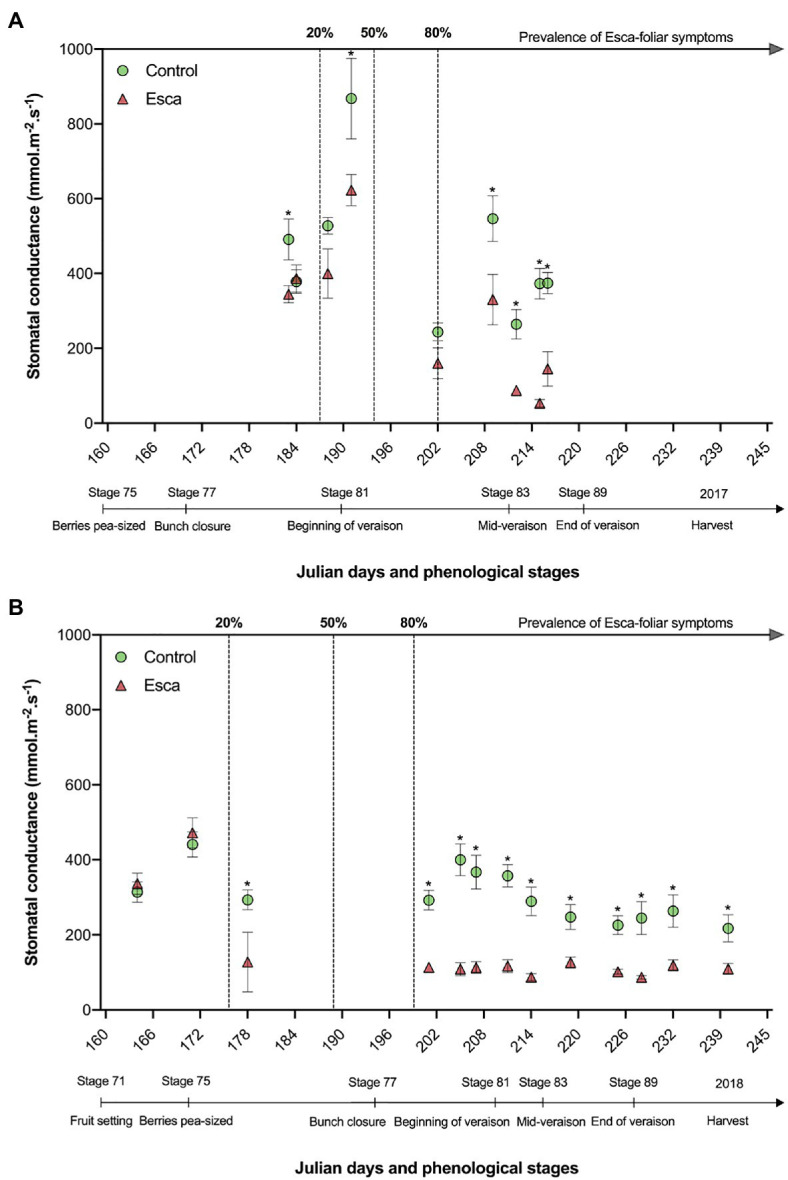
Evolution of stomatal conductance (g_s_) in asymptomatic and symptomatic Cabernet Sauvignon grapevines recorded in 2017 **(A)**, and in 2018 **(B)**. Key-growing phenological stages and the prevalence of Esca appearance are also represented below and above the horizonal axes, respectively. Error bars represent SE, and stars above g_s_ values at a given date indicate a significant difference between asymptomatic and symptomatic grapevines at *p* < 0.05.

When grapevines had not yet expressed any Esca-foliar symptoms (prevalence of symptoms <20%) in 2017 and 2018, no conclusive and consistent differences in stomatal conductance (g_s_) were measured (*p* > 0.05). However, when the prevalence of Esca-foliar symptoms reached 50 to 80%, corresponding to stage 81 on the BBCH scale for 2017 and stage 75 for 2018, the average g_s_ dropped significantly (*p* = 0.05 and *p* = 0.02 in 2017 and 2018, respectively) in Esca-diseased grapevines compared with control plants. In both years, the average g_s_ measured on the leaves that progressively became Esca-symptomatic during veraison was one point five times lower than in control leaves (Figure 5). When the prevalence of Esca-foliar symptoms reached 80%, and most of Esca-foliar symptoms had appeared, g_s_ of Esca-symptomatic leaves rarely exceeded 200 mmol.m^−2^.s^−1^, while g_s_ in control leaves remained at around 300 mmol.m^−2^.s^−1^. After stage 83 (mid-veraison), Esca-diseased leaves started to significantly dry and curl, preventing accurate measurement of g_s_. This difference was also recorded in the earlier stages, when the prevalence of Esca-foliar symptoms had just reached 20% and when most leaves were in a presymptomatic state.

**Figure 6:**
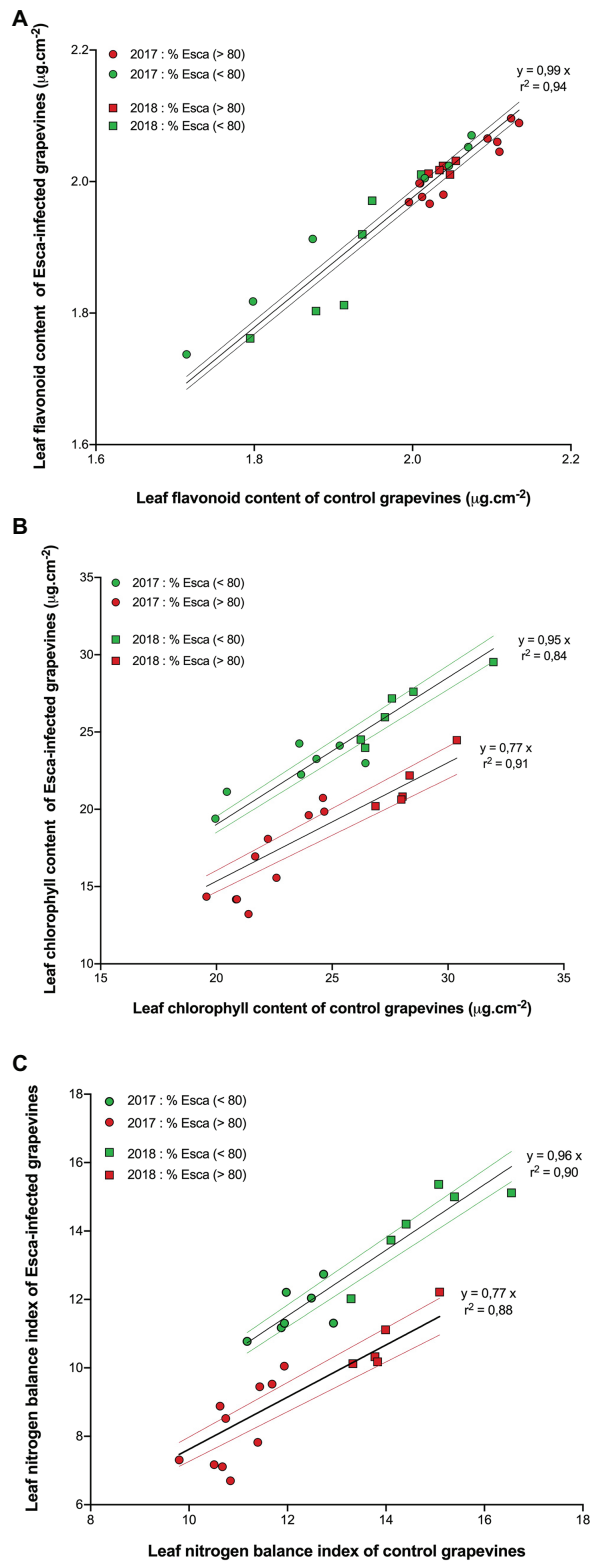
Responses of **(A)** leaf flavonoid **(B)** chlorophyll content, and **(C)** leaf nitrogen index in pre-Esca-symptomatic and Esca-symptomatic leaves in comparison with control (healthy) leaves. Data represent the average of 64 leaves for each modality. Data were separated to reflect days “before” (green symbols) and “after” (red symbols) prevalence of Esca-foliar symptoms reached 80% in 2017 (circles) and 2018 (squares). Regression models (full lines) and a 95% confidence interval (colored-dotted lines) are also indicated.

The responses of flavonols (Flav), chlorophyll (Chl), and of the nitrogen balance index (NBI) content in Esca-symptomatic grapevines and control plants are presented in Figure 6. There was no difference (*p* > 0.5) in Flav content regardless of the year of measurement or the onset of Esca-foliar symptoms between control and Esca-symptomatic leaves (Figure 6A). However, Chl and NBI were significantly lower in Esca-symptomatic leaves when the prevalence of Esca-leaf symptoms reached the 80% threshold; on average, once most symptoms had appeared on Esca-diseased grapevines, Chl and NBI measurements were 23% lower on symptomatic leaves compared with control ones (Figures 6B,C). As foliar symptoms spread on Esca-symptomatic grapevines, chlorophyll activity (and thus photosynthetic functions) was progressively altered, even in the apparently healthy and green parts of the Esca-symptomatic leaves. Regardless of the onset of Esca-foliar symptoms, the year of measurement had an effect on the level of expression of both Chl and NBI indices as data collected in 2018 presented higher values (*p* < 0.05) compared with 2017 (Figures 6B,C).

**Figure 7:**
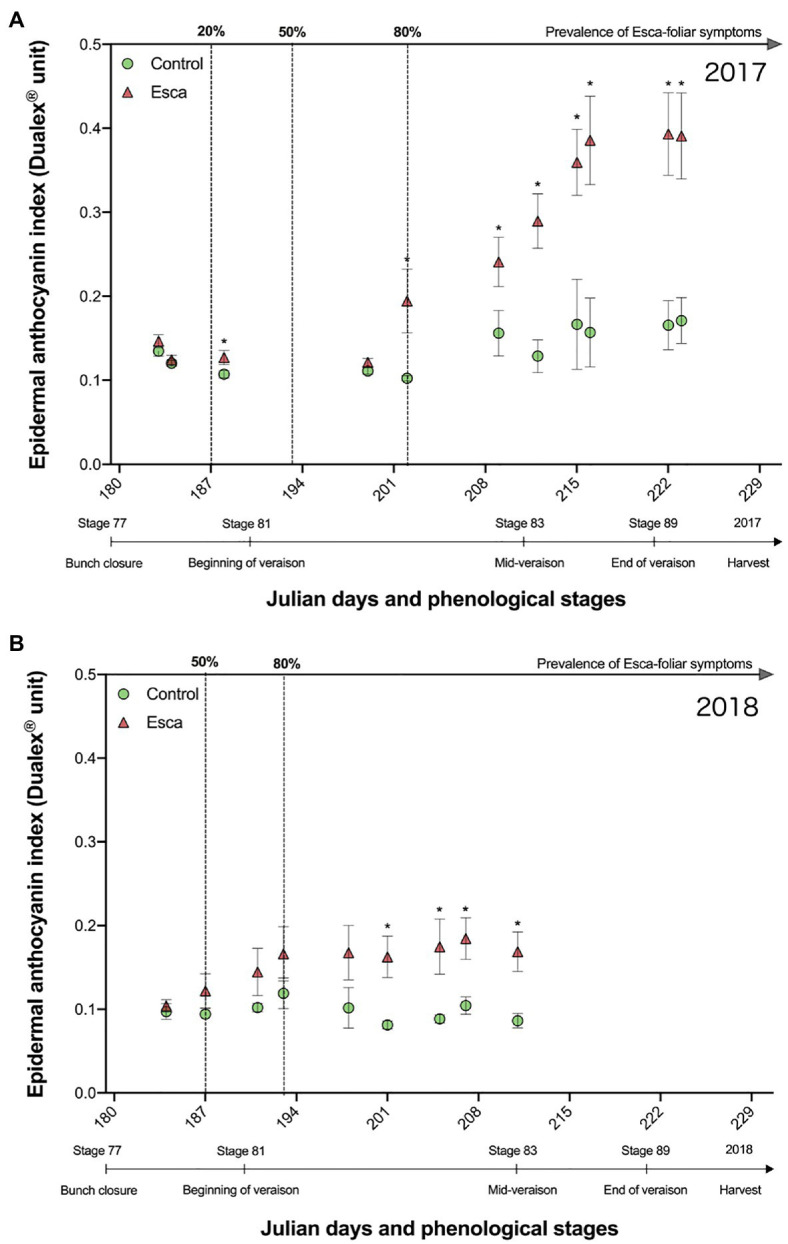
Responses of anthocyanins in presymptomatic and Esca-symptomatic leaves in comparison with control leaves measured in 2017 **(A)** and in 2018 **(B)**. Key-growing phenological stages and the prevalence of Esca appearance are also represented below and above the horizonal axes, respectively. Error bars represent SE, and a star for a given date indicates a significant difference between asymptomatic and symptomatic grapevines at *p* < 0.05.

The evolution of anthocyanin (Anth) content in control and Esca-symptomatic leaves was consistent in 2017 and 2018 (Figure 7). Once most Esca-foliar symptoms had appeared on infected grapevines, Anth content in Esca-symptomatic leaves was significantly higher (*p* < 0.05) compated with controls (Figure 7). In healthy leaves, values remained stable throughout the season. However, in Esca-symptomatic leaves, the Anth content during veraison seemed to increase, especially in 2017, where values were two times as high as in control leaves (Figure 7A).

### Esca Emergence and Gene Expressions of Primary Physiological Functions

**Figure 8:**
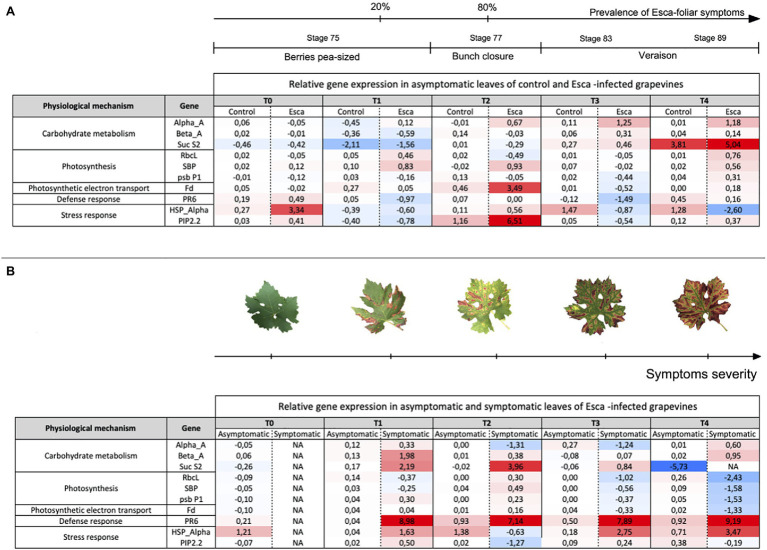
“Heatmap” representing the relative expression levels of 10 targeted genes (in Log 2 transformations) involved in carbohydrate metabolism (three genes), photosynthesis (three genes), photosynthetic electron transport (one gene), defense mechanism (one gene), and stress responses (two genes). **(A)** Comparison of genes expression levels in asymptomatic leaves sampled from either control or Esca-diseased grapevines, and **(B)** comparison of genes expression levels in asymptomatic and symptomatic leaves sampled from grapevines that expressed Esca-foliar symptoms. A blue-color gradient represents genes that were repressed (Log 2 (RE) < 0); a red-color gradient represents genes that were over-expressed (Log 2 (RE) > 0), and a white-color gradient represents genes exhibiting no modification in their expression (Log 2 (RE) = 0). Values are means ± SD of two independent biological replicates. Key-growing phenological stages, leaf symptom severity, and the prevalence of Esca appearance are also represented.

Targeted genes involved in primary metabolism and physiological regulatory functions were expressed differently, depending on the grapevine phenological stage and the presence or absence of Esca-foliar symptoms (Figure 8). RT-qPCR profiles of genes involved in starch degradation, stress response, and a defense mechanism showed clear discrimination between healthy and Esca-symptomatic leaves. In asymptomatic leaves of control and Esca-diseased grapevines, most of the targeted genes were firstly downregulated (T1) and finally unchanged or upregulated, except for HSP-α in asymptomatic leaves of Esca-affected vines (Figure 8A). When the prevalence of Esca-foliar symptoms reached 20% (T1), at growing stage 75, PR6 and Beta-A were significantly upregulated in symptomatic leaves (Figure 8B). HSP-α was also upregulated in symptomatic leaves, especially during veraison at stages 83 and 89. The induction of those genes could be explained by the progressive spread of Esca-foliar symptoms on sampled leaves, leading to severe disruption of photosynthetic-related functions, showed notably by a downregulation of RbcL, SBP, and psbP1 (Figure 8B). As a matter of fact, the Esca-symptoms severity of sampled leaves gradually increased, each time leaves were collected for RT-qPCR analysis (Figure 8B).

**Figure 9:**
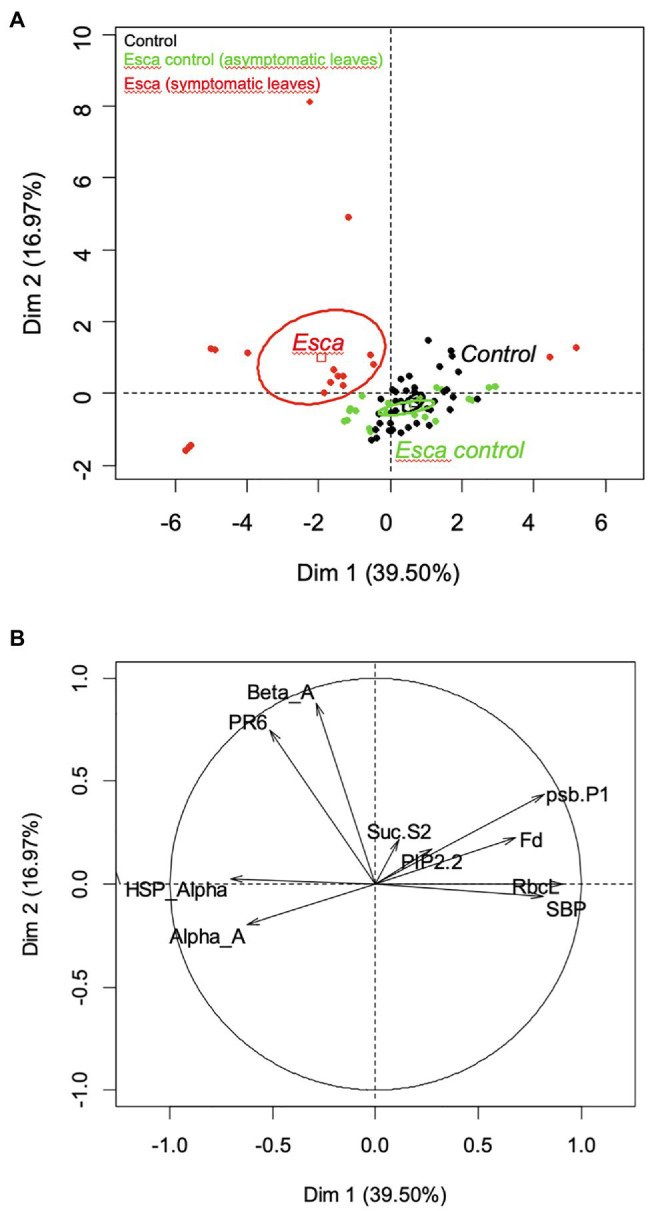
Principal component analysis (PCA) for the quantitative reverse transcription (RT-qPCR) results, showing **(A)** the confidence ellipses for control (black), Esca-asymptomatic (green), and Esca-symptomatic (red) leaf samples, and **(B)** the correlation circle with the two axes of PCA performed for control, Esca-asymptomatic, and Esca-symptomatic leaf samples.

The principal component analysis revealed that comparison between symptomatic and healthy leaves from Esca-diseased vines explained around 60% of the differences, whereas comparison between healthy leaves sampled on control plants or Esca-diseased grapevines accounted for 40% of observed differences (data not shown). Analysis of the gene expression profiles of Esca-diseased grapevines confirmed the strong downregulation of photosynthesis-related genes (psbP1, Fd, RbcL, SBP) in Esca-symptomatic leaves during veraison (T3 and T4, Figures 8B, 9). Esca-symptomatic leaves sampled during the previous phenological stages did not show a similar repression (T1 and T2), despite the presence of some leaf discolorations. In addition, the expression of genes involved in stress response (HSP-α) and defense mechanisms in response to pathogen infection (PR6) was also upregulated once the 80% threshold of Esca-foliar symptoms prevalence (T3 and T4) was reached. Control leaves sampled from both control and Esca-symptomatic grapevines did not show significant differences (*p* > 0.1) in gene-level expressions. The different sampling times and the corresponding increase severity of Esca-foliar symptoms had no impact on the qPCR profiles of control leaves collected from Esca-symptomatic grapevines.

## Discussion

In our experiment, we observed that discrimination between healthy and Esca-diseased grapevines based on measurements of foliar physiological activities or chemical compounds was not entirely relevant because significant differences were only observed concurrently or after the expression of the first foliar symptoms in the plant. On the contrary, grapevine sap flow measurements were significantly lower in Esca-diseased plants several weeks before changes in the expression of leaf genes, such as stress- and defense-genes were observed, and the appearance of any foliar symptoms. These findings are discussed in detail hereafter.

The onset of Esca-foliar symptoms happened earlier in 2017 than in 2018. As reported by Surico et al. (2006), Bruez et al. (2013), and Songy et al. (2019), climate variability can influence Esca foliar expression. We hypothesize that higher summer temperatures and precipitations in 2018 than in 2017 could potentially explain the earlier Esca-symptomatic expression that year, which is in line with observations from Surico et al. (2000) and Lecomte et al. (2012). Additionally, the highest values of transpiration were observed in 2018, before the onset of Esca-foliar symptoms, which were certainly due to fully saturated soil in spring and the development of larger LAI.

Image analyses performed on the longitudinal sections of trunks and cordons at the end of the monitoring period revealed the severity level of internal wood necroses. It was particularly clear that a white rot was always present in plants that expressed Esca-foliar symptoms. The quantity of white rots in the cordons is also a parameter to be considered with a prescribed threshold value of at least 10–20% (Figure 2) being a strong descriptor for the chronic form of Esca (Ouadi et al., 2019; Songy et al., 2019).

Regardless of the date of appearance of Esca-foliar symptoms, whole-plant transpiration was systematically about 40 to 50% lower in Esca-diseased grapevines compared with controls. It should be noted that because no difference in LAI between symptomatic and asymptomatic plants was shown (Supplementary Figure S1), patterns in transpiration found on a leaf-area basis were similar to those on a ground-area basis. The lack of difference in the leaf area between treatments was likely due to the management practices applied to the whole vineyard that involved uniform mechanical leaf removal just after veraison and just before harvest. The effect of extensive leaf pruning on day 230 is, for example, visible in Supplementary Figure S1B and marked by a decline in transpiration, but, even after this cultural practice, the difference in transpiration rates between asymptomatic and symptomatic vines remained significant.

The decrease in transpiration in Esca-diseased grapevines was observed at least 2 weeks before any Esca-foliar symptoms appeared, and this difference in transpiration was not affected by climatic conditions (air temperature or atmospheric VPD). However, higher levels of transpiration were recorded for both Esca-symptomatic and control grapevines when VPD was particularly strong (>3.5 kPa; Supplementary Figures S2B,C). On the contrary, the decline in midday sap flow under dry climatic conditions in the Esca-diseased grapevines reflected the decrease in stomatal conductance and may indicate that soil and roots became more disconnected as the soil dried, or that a root, physiologically, was also directly impacted by Esca. Prior to the onset of apparent Esca-foliar symptoms, the continuous monitoring of sap flow indicated a decrease in water transport within Esca-diseased grapevines. Because there was a large amount of wood necroses, including in the sapwood tissue, it can be suggested that the vascular system was altered (Maher et al., 2012), and that the loss of grapevine water transport capacity resulted from these vessel occlusions or destructions (McElrone et al., 2010). Additionally, the formation of tyloses and gummosis can occur within vessels of the leaves as recently demonstrated by Bortolami et al. (2019, 2021), thus, decreasing even further the permeability of functional vessels.

Dynamic changes in tissue water content over space and time can impact the interpretation of plant water use and how various compartments contribute to an integrated response to plant stress. Stem water potential or daily stem diameter changes measured with dendrometers are commonly used for estimating grapevine water status in the field (Chone et al., 2001; Ahiman et al., 2017). However, those measurements are time-consuming or need constant field maintenance, which makes them impractical for daily routines. In the present study, thermal sap flow sensors were employed to assess whole plant water use, which responded with high precision to climatic variations and integrated the complex interactions that existed between grapevine functioning, grapevine structure, and soil properties. In addition to environmental factors, those sensors can also potentially reflect the way grapevines are managed (i.e., thinning, irrigation), which is particularly relevant with a perennial crop. Because it is a fundamental plant-based index, we suggest that sap flow variations analysis can be used to make practical decisions related to Esca-foliar symptomatology and grapevine performance in a context of climate change prediction. Methods allowing the direct observation of the xylem loss of hydraulic conductivity, i.e., an embolism, in plants, such as magnetic resonance imaging (MRI) and X-ray micro computed tomography (microCT), represent other reliable options to visualize vascularization dysfunctions induced by Esca-diseased grapevines (Choat et al., 2010; Knipfer et al., 2016). These techniques have provided new insights into the structure and function of plant hydraulic networks and the stomatal control of leaf loss of hydraulic conductivity (Hochberg et al., 2016). To improve our understanding and interpretation of datasets captured with sap flow methods, those imaging measurements should be considered along with stem water use when characterizing Esca-foliar symptoms and their consequences on whole-plant structural and functional acclimations to the infection.

Our study revealed that Esca emergence leads to significant disturbances of stomatal conductance (g_s_), leaf metabolism, and changes in different phenolic families at the early stage of Esca-symptoms expression. According to Petit et al. (2006) and Magnin-Robert et al. (2011), both stomatal closure and alteration of the photosynthetic machinery are observed in Esca-symptomatic leaves, but the physiological mechanisms associated with the appearance of leaf symptoms have not been yet clearly determined. In those studies, all measurements were performed on both healthy and Esca-symptomatic leaves, and, unlike in your study, no recurrent differences in stomatal conductance were recorded between asymptomatic leaves of Esca-diseased grapevines and controls, only a decrease in total chlorophyll content and in the efficiency of the photosystem II was observed in presymptomatic Esca-leaves of symptomatic plants (Petit et al., 2006; Magnin-Robert et al., 2011).

In viticulture, it is important to estimate grapevine nitrogen availability as it affects yields, influences grape fermentation potential, and defines grape quality *via* phenolic activity during maturation (Soubeyrand et al., 2014; Cerovic et al., 2015). In plants, nitrogen content and also phenolic compounds and chlorophyll contents influence stomatal regulation and photosynthetic activity (Farquhar et al., 2002; Louis et al., 2009). Our study revealed that epidermal phenolic compounds and NBI profiles were significantly lower in Esca-symptomatic leaves, indicating that leaf nitrogen availability was also lower in Esca-affected leaves, and, thus, that the whole photosynthetic machinery was negatively impacted. In addition, the 2-year survey period confirmed that photosynthetic pigments were severely affected by Esca as Chl significantly decreased with the outbreak of foliar symptoms, but that Anth content increased, which is usually indicative of a plant stress (Guerin-Dubrana et al., 2019). In line with this result, Martín et al. (2019) have recently observed that chlorophylls and carotenoids contents and some alterations on their phenolic profiles were reduced in Esca-symptomatic leaves of Tempranillo cultivar.

Confirming patterns in epidermal phenolic compounds and NBI profiles of Esca-symptomatic leaves, drastic alterations of photosynthetic functions were also observed through activation of specific gene responses. Activities of certain genes such as Fd, RbcL, SBP, and psb P1 were coherent with the repression of photosynthetic genes observed in Esca-diseased Chardonnay grapevines (Magnin-Robert et al., 2011). In addition, Esca-symptomatic leaves over-expressed the defense-related gene PR6 at an early symptomatic stage, which was consistent with several studies that described an upregulation of pathogenesis-related proteins in Esca-symptomatic leaves (Letousey et al., 2010; Magnin-Robert et al., 2011). Regarding asymptomatic leaves that were sampled on both Esca-diseased and healthy-control grapevines, our results revealed a general downregulation of stress response proteins (HSP) simultaneously at the onset of Esca leaf symptoms (from berries, pea sized, to the end of veraison). The protection of cellular functions can be provided by small chaperone heat-shock proteins, which have been reported in the brown stripe, a typical wood discoloration attributed to another GTD, Botryosphaeria dieback (Spagnolo et al., 2014; Magnin-Robert et al., 2016). Our results support the hypothesis that HSPs could then be related to some cellular dysfunctions associated with the expression of Esca symptoms (Magnin-Robert et al., 2016) and could be involved in a plant resistance mechanism (Yang et al., 2011).

In the meantime, an overall upregulation of Alpha-A proteins (amylases) was measured in asymptomatic leaves of Esca-diseased plants simultaneously to Esca leaf symptoms appearance from bunch closure to veraison. A similar but less pronounced trend was found regarding the regulation of Beta-A proteins. The occurrence of a higher starch grain content in asymptomatic leaves from healthy canes rather than those from infected canes can be considered as a first defense step (Troccoli et al., 2001; Valtaud et al., 2009). The high amylase activity recorded in asymptomatic leaves sampled on Esca symptomatic grapevines was likely linked to an increase in starch hydrolysis and to a higher production of glucose and fructose (Valtaud et al., 2011). This result supports the hypothesis that high content of fructose in Esca-diseased leaves would be involved in a detoxification process rather than in the nutrition of wood-inhabiting fungi, as fructose is not preferentially used by Esca pathogens (Crecelius et al., 2003; Luini, 2007).

To conclude, grapevine sap flow measurements were significantly lower in Esca-diseased plants weeks before any changes appeared in the leaves. Unlike grapevine sap flow disruption, structural (e.g., leaf discolorations), functional (e.g., stomatal conductance, photosynthetic activity, phenolic compounds), and genetic (e.g., the expression of leaf- targeted genes) plant responses were only significantly impacted by Esca at the onset and during the leaf symptoms development. Reduced sap flow dynamic, which was related to a high level of a white-rot necrosis, provided a useful tool to early predict internal plant disorders due to Esca-grapevine disease.

## Data Availability Statement

The original contributions presented in the study are included in the article/Supplementary Material, further inquiries can be directed to the corresponding author.

## Author Contributions

LO, J-CD, EB, FF, CC, AY, and PR designed the experiments and/or analyzed the data. LG-D and SB provided the data on Esca-disease history of the plants used in this study. LO, EB, and SB participated in the various measurement campaigns in the vineyard (e.g., sap flow, leaf stomatal conductance, photosynthesis, and phenolic activities) and in the sap flow sensors maintenance. LO measured the wood necrosis. LO, FF, and CC participated in the gene expression experiment. LO, J-CD, PR, EB, and FF wrote the manuscript. All authors contributed to the article and approved the submitted version.

### Conflict of Interest

The authors declare that the research was conducted in the absence of any commercial or financial relationships that could be construed as a potential conflict of interest.

## Footnotes

^1^https://imagej.nih.gov/ij/

## Supplementary Material

Supplementary Figure S1 Evolution of leaf area index (LAI in m^2^ of leaves per m^2^ of ground) in asymptomatic and symptomatic grapevines during the 2017 and 2018 growing seasons. Large symbols in the middle of each panel represent overall stand means (± SE) of asymptomatic and symptomatic grapevines.

Supplementary Figure S2 Evolution of sap flow in asymptomatic and symptomatic grapevines under different climatic conditions. **(A)** Diurnal courses of sap flow in asymptomatic and symptomatic grapevines recorded in 2017 (right) and 2018 (left). **(B)** Evolution of temperature and vapor-pressure deficit (VPD) conditions in 2017 (right) and 2018 (left). Error bars in panel **(A)** represent SE.
