# Supplementary figures and images for: Sap Flow Disruption in Grapevine Is the Early Signal Predicting the Structural, Functional, and Genetic Responses to Esca Disease
Source: Front Plant Sci. 2021 Jul 1;12:695846. doi: 10.3389/fpls.2021.695846 (PMC8281252; doi:10.3389/fpls.2021.695846)

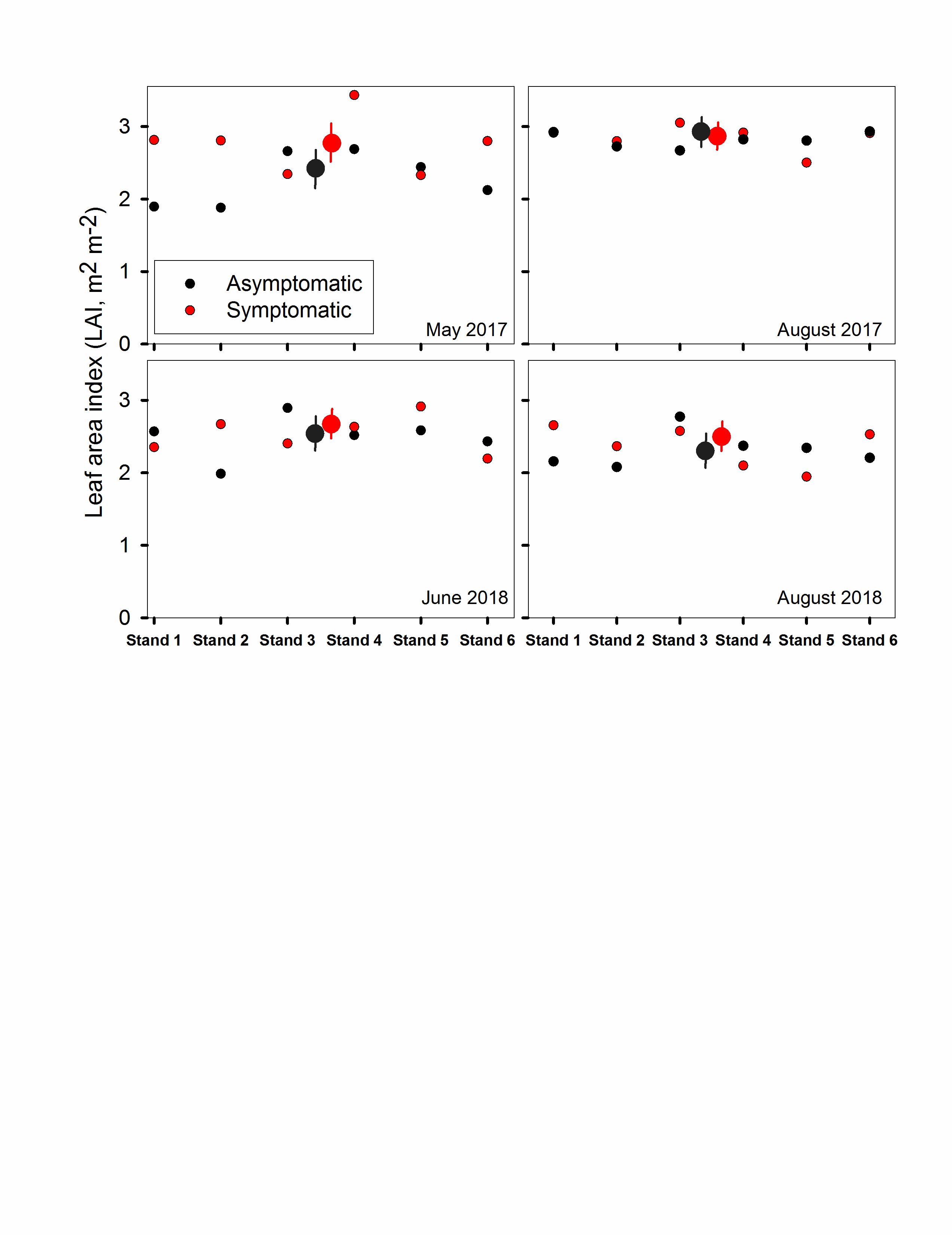

Supplement: Supplementary Figure S1 — Evolution of leaf area index (LAI in m2 of leaves per m2 of ground) in asymptomatic and symptomatic grapevines during the 2017 and 2018 growing seasons. Large symbols in the middle of each panel represent overall stand means (± SE) of asymptomatic and symptomatic grapevines. [file Image_1.TIF]

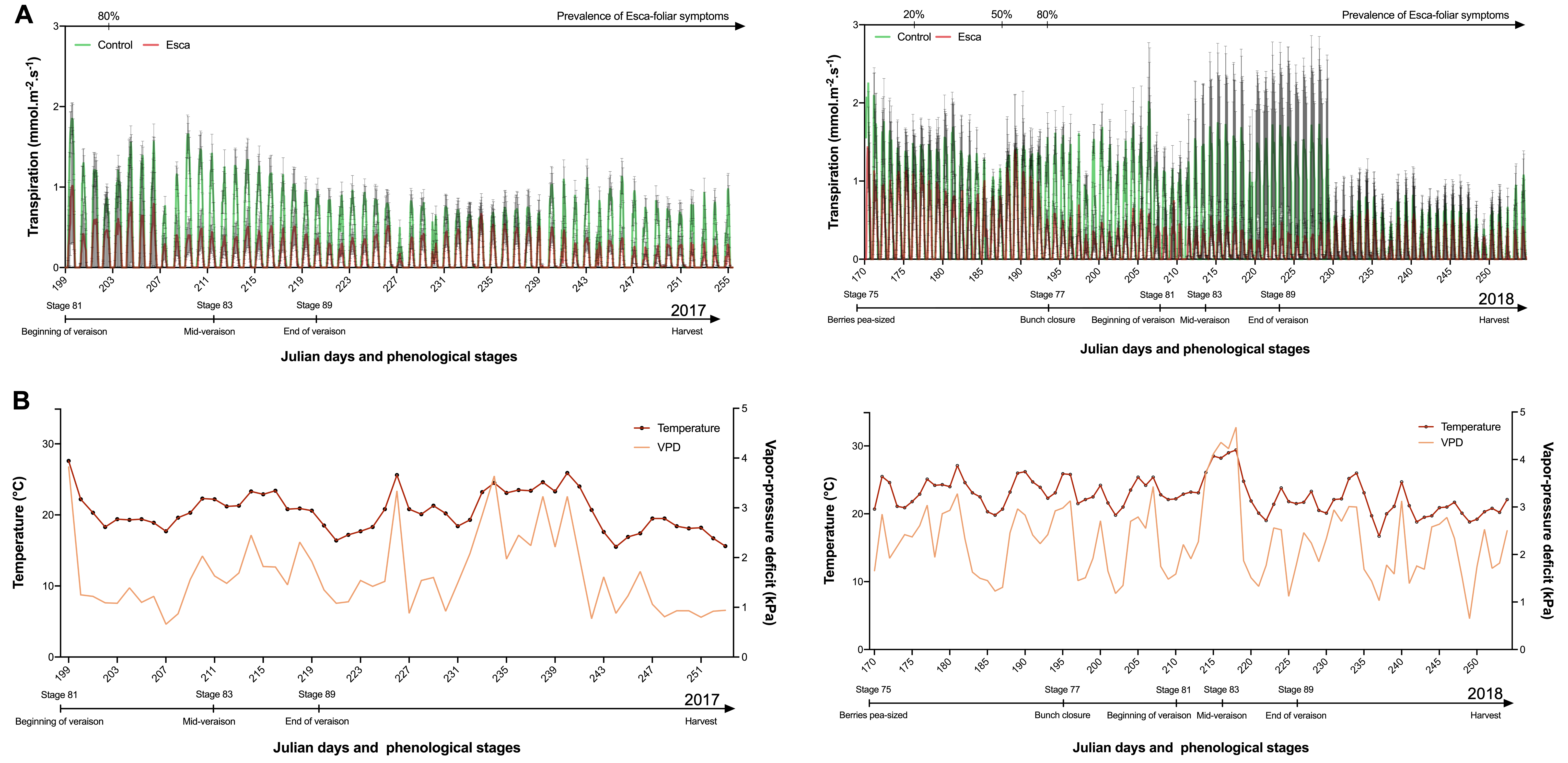

Supplement: Supplementary Figure S2 — Evolution of sap flow in asymptomatic and symptomatic grapevines under different climatic conditions. (A) Diurnal courses of sap flow in asymptomatic and symptomatic grapevines recorded in 2017 (right) and 2018 (left). (B) Evolution of temperature and vapor-pressure deficit (VPD) conditions in 2017 (right) and 2018 (left). Error bars in panel (A) represent SE. [file Data_Sheet_2.ZIP › Fig S2.tif]
